# SAGES: A Suite of Freely-Available Software Tools for Electronic Disease Surveillance in Resource-Limited Settings

**DOI:** 10.1371/journal.pone.0019750

**Published:** 2011-05-10

**Authors:** Sheri L. Lewis, Brian H. Feighner, Wayne A. Loschen, Richard A. Wojcik, Joseph F. Skora, Jacqueline S. Coberly, David L. Blazes

**Affiliations:** 1 National Security Technology Department, The Johns Hopkins University Applied Physics Laboratory, Laurel, Maryland, United States of America; 2 Division of GEIS Operations, Armed Forces Health Surveillance Center, Silver Spring, Maryland, United States of America; Kenya Medical Research Institute - Wellcome Trust Research Programme, Kenya

## Abstract

Public health surveillance is undergoing a revolution driven by advances in the field of information technology. Many countries have experienced vast improvements in the collection, ingestion, analysis, visualization, and dissemination of public health data. Resource-limited countries have lagged behind due to challenges in information technology infrastructure, public health resources, and the costs of proprietary software. The *Suite for Automated Global Electronic bioSurveillance* (SAGES) is a collection of modular, flexible, freely-available software tools for electronic disease surveillance in resource-limited settings. One or more SAGES tools may be used in concert with existing surveillance applications or the SAGES tools may be used *en masse* for an end-to-end biosurveillance capability. This flexibility allows for the development of an inexpensive, customized, and sustainable disease surveillance system. The ability to rapidly assess anomalous disease activity may lead to more efficient use of limited resources and better compliance with World Health Organization International Health Regulations.

## Introduction

### History and Background

Emerging and re-emerging infectious diseases are a serious threat to global public health. [1], [2] The World Health Organization (WHO) has identified more than 1100 epidemic events worldwide in the last five years alone. [3] Recently, the emergence of the novel 2009 influenza A (H1N1) virus and the SARS coronavirus have demonstrated how rapidly pathogens can spread worldwide. [4] This infectious disease threat, combined with a concern over man-made biological or chemical events, spurred WHO to update their International Health Regulations (IHR) in 2005. [5] The new 2005 IHR, a legally binding instrument for all 194 WHO member countries, significantly expanded the scope of reportable conditions and are intended to help prevent and respond to global public health threats. SAGES, an electronic biosurveillance initiative described herein, aims to improve local public health surveillance and IHR compliance with particular emphasis on resource-limited settings.

Electronic disease surveillance, particularly syndromic surveillance, holds promise to improve health security in resource-limited environments. [6], [7] Such systems have become versatile tools in health departments in the United States. [8] Epidemiologists using electronic disease surveillance not only have the potential to detect anomalous disease activity earlier than traditional laboratory-based surveillance, but they also have the ability to monitor the health of their community in the face of a known threat. [9], [10], [11] More than a decade ago, in collaboration with the US Department of Defense (DoD), the Johns Hopkins University Applied Physics Laboratory (JHU/APL) developed the *Electronic Surveillance System for the Early Notification of Community-based Epidemics* (ESSENCE). ESSENCE collects, processes, and analyzes non-traditional data sources (i.e. chief complaints from hospital emergency departments, school absentee data, poison control center calls, over-the-counter pharmaceutical sales, etc) to identify anomalous disease activity in a community. The data can be queried, analyzed, and visualized both temporally and spatially by the end user. [10] ESSENCE is currently being utilized by the US Department of Defense, US Veterans Health Administration, and numerous state and local health departments in the US. [11]

The current SAGES initiative leverages the experience gained in the development of ESSENCE, and the analysis and visualization components of SAGES are built with the same features in mind. This paper will describe the key features and potential uses of the SAGES product in developing or resource-limited settings.

## Methods

### Selected Advances - SAGES

The Global Emerging Infections Surveillance and Response System, a division of the Armed Forces Health Surveillance Center, is committed to enhancing electronic disease surveillance capacity in resource-limited settings around the world. To this end, they have entered into a robust collaboration with the JHU/APL to create SAGES. Cognizant of work underway on individual surveillance systems components, e.g., collection of data by cell phones, we have focused our efforts on the integration of inexpensive and/or freely available, interoperable software tools that facilitate regional public health collaborations. [12]

**Figure 1:**
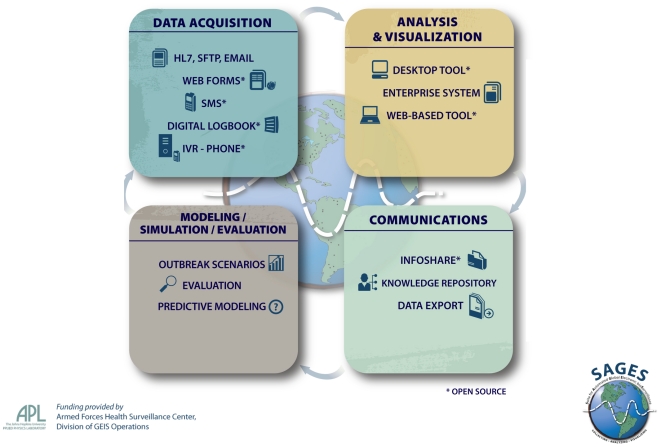
SAGES Suite of Tools. The *Suite for Automated Global Electronic bioSurveillance* (SAGES) is a collection of modular, flexible, freely-available software tools for electronic disease surveillance in resource-limited settings. One or more SAGES tools may be used in concert with existing surveillance applications or the SAGES tools may be used *en masse* for an end–to-end biosurveillance capability.

SAGES tools are organized into four categories: 1) data collection, 2) analysis & visualization, 3) communications, and 4) modeling/simulation/evaluation. (Figure 1) Within each category, SAGES offers a variety of tools compatible with surveillance needs and different types or levels of information technology infrastructure. In addition to the flexibility of tool selection, there is flexibility in the sense that the analysis tools do not require a fixed database format. For example, rather than requiring an existing database to adapt to the tool, the SAGES database tools adapt to the format of all Java database compliant formats. Lastly, the SAGES tools are built in a modular nature, which allows for the user to select one or more tools to enhance an existing surveillance system or use the tools *en masse* for an end-to-end electronic disease surveillance capability. Thus, each locality can select tools from SAGES based upon their needs, capabilities, and existing systems to create a customized electronic disease surveillance system.

#### Data Acquisition

Rapid data acquisition is arguably the most challenging aspect of establishing a successful electronic disease surveillance system. [9], [11] In resource-limited settings, it is imperative to select the technology that is both easy to incorporate into existing health services and sustainable with little or no additional financial investment. The approach should allow customizable data collection, enable multiple data streams collected in different ways, and be scalable based upon needs. [6], [7] Data collection tools included within SAGES are web forms, short message service texting programs, digital logbooks, and interactive voice response systems. Data entered into SAGES can be validated by the stakeholder. Where appropriate, other collection methods such as email and secure file transfer protocol can be applied as well. The data acquisition methods in SAGES are readily adaptable to evolving standards in both minimum data sets for disease surveillance and routine diagnosis and care.

#### Analysis & Visualization

As previously discussed, the SAGES analysis and visualization tools are built upon the features and functionality of the more mature enterprise ESSENCE system. The enterprise ESSENCE system requires a high speed internet connection, relies on automated data streams, and uses proprietary software for the display of data. [10], [11], [13] The SAGES web-based application and the desktop application are both freely-available tools that provide similar functionality to countries with limited public health resources. All ESSENCE applications contain alerting algorithms developed by JHU/APL and the Early Aberration Reporting System (EARS) algorithms developed by the U.S. Centers for Disease Control and Prevention (CDC) to identify anomalous events. Users also have the ability to add additional algorithms as desired.

**Figure 2:**
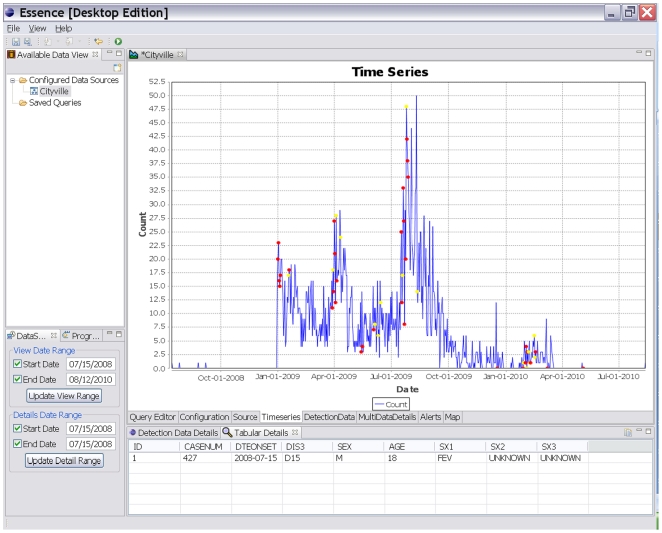
Times Series Display. Through *ESSENCE Desktop Edition (EDE)*, users have the ability to see time series views of their data labeled with early event detections. Detections are generated using one of the default algorithms supplied with EDE; however, users can add additional algorithms of their choosing.

**Figure 3:**
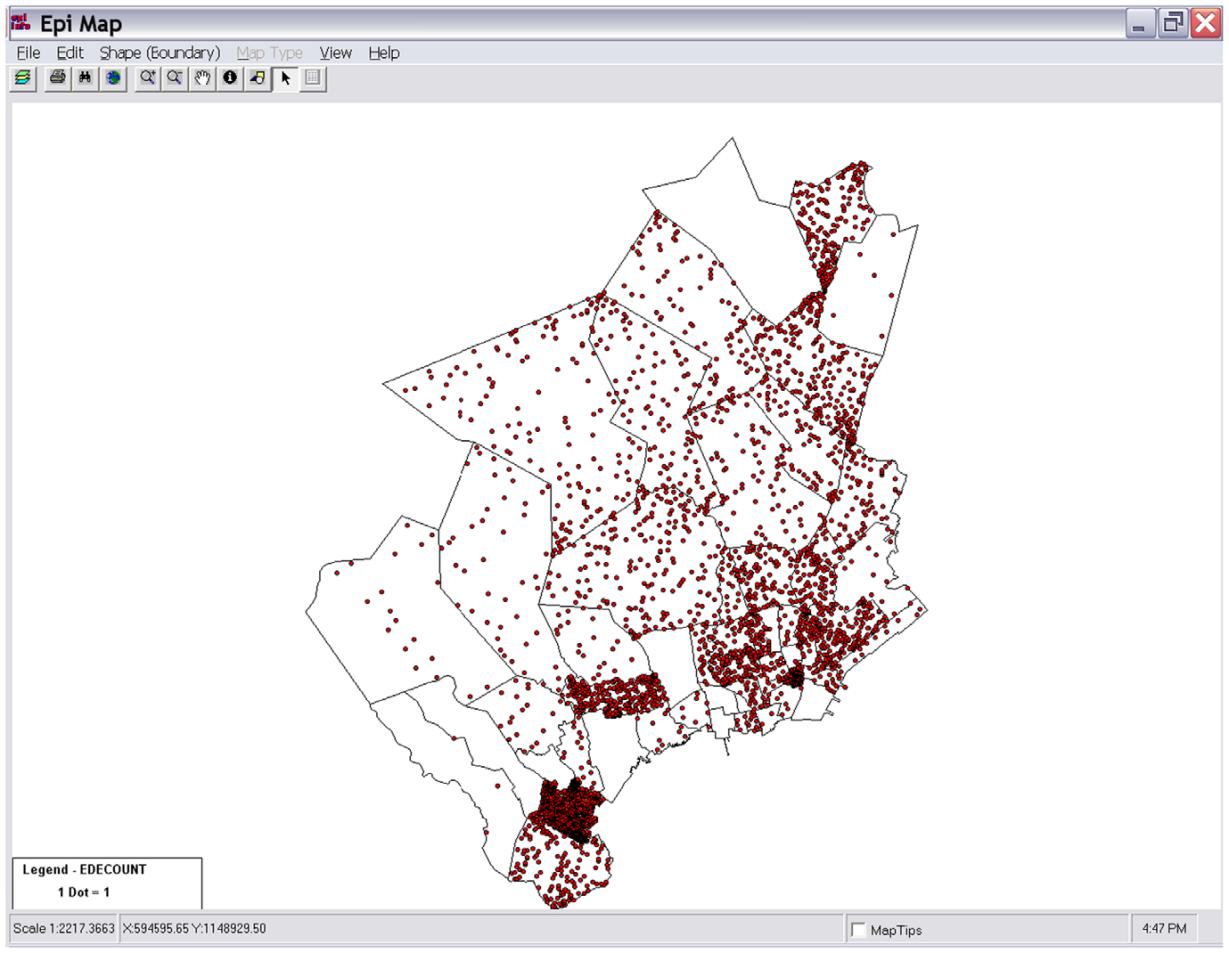
Mapping Utilities. *OpenESSENCE* and EDE users can map spatial data, cases, and alert levels in geographic areas defined in the data. OpenESSENCE embeds maps in the application using a GeoServer interface. EDE embeds maps through a uDig interface or optionally launches CDC's EpiMap application with Environmental Systems Research Institute shape files.

**Figure 4:**
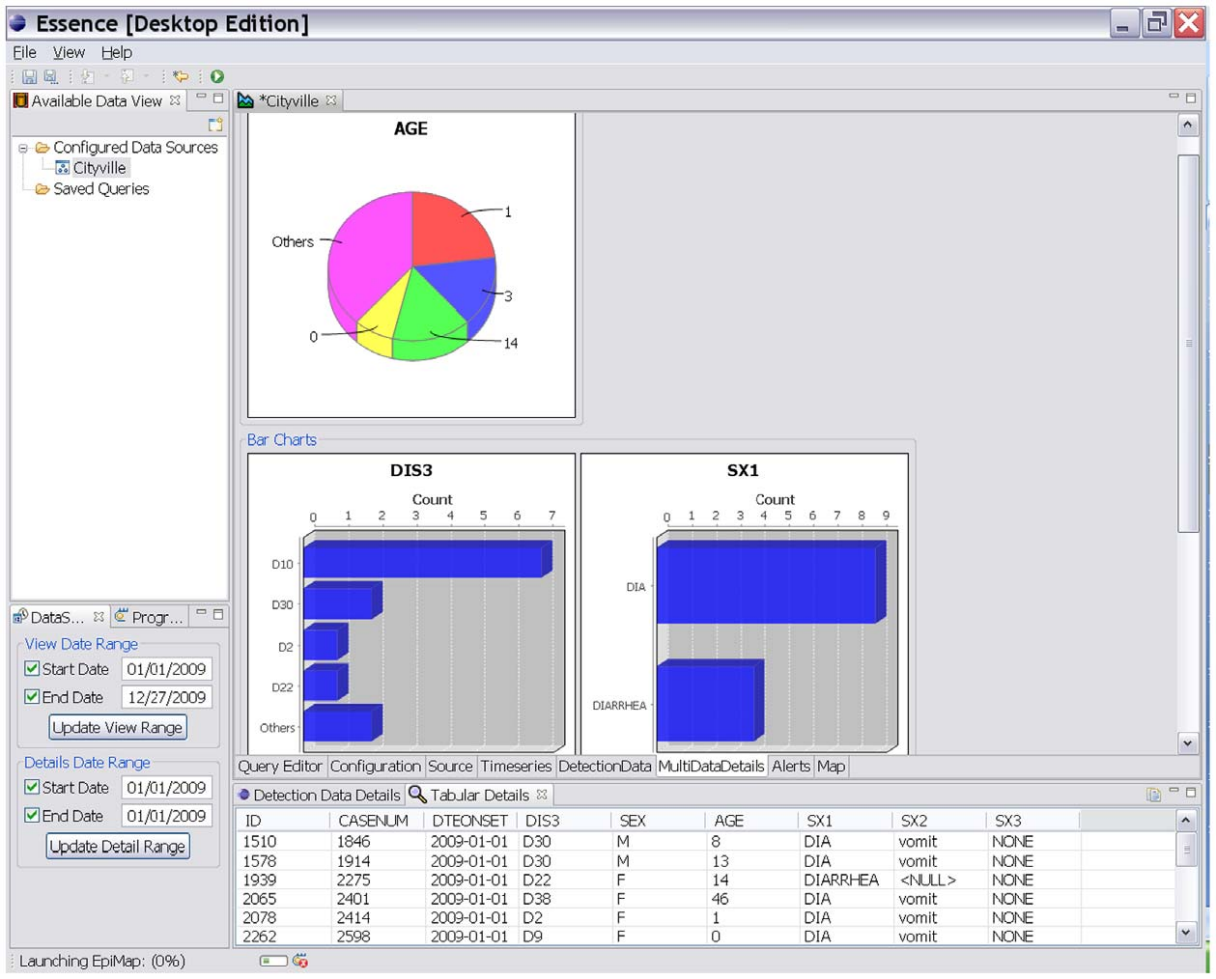
Chart and Graph Utilities. Open ESSENCE and EDE both have various tools for the visualization of data. All graphs and charts products may be exported into common graphic formats.

The desktop tool, known as ESSENCE Desktop Edition (EDE), is a single-user stand-alone analysis and visualization tool that can be installed on most computers. EDE does not need access to the internet as it ingests data files stored on the same computer. The web-based tool, known as OpenESSENCE, is a multi-user network accessible data entry, analysis, and visualization tool that enables an epidemiologist to monitor the population's health from any computer connected to that network. Available analyses for both tools depend on the nature of the data ingestion, but may include demographic characterizations, temporal and spatial analyses, display of patient level information, geographic information system mapping, anomalous event detection, and dynamic query capability. (Figures 2, 3, 4)

#### Communications

**Figure 5:**
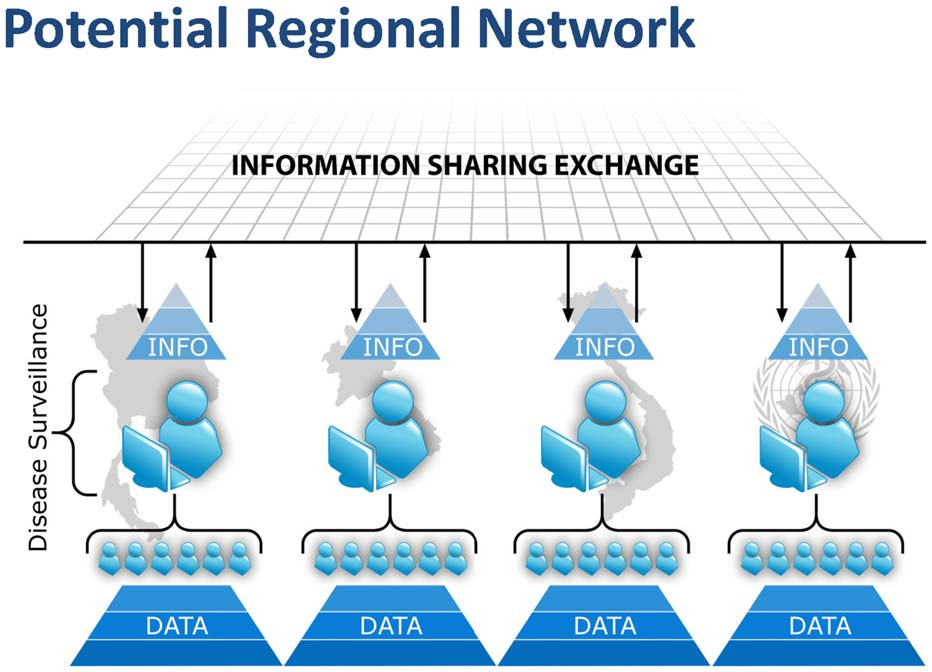
Role-based Information Sharing. InfoShare allows each user to determine role-based access to public health information. The data are collected and stored only by the user, and remain under the sole control of the user at all times.

SAGES tools can facilitate compliance with 2005 IHR reporting requirements and allow the sharing of actionable information across jurisdictional boundaries. Sharing of patient-level data across regional boundaries is generally not realistic and often not helpful, as local public health entities are usually best suited to interpret local events. However, once these data have been transformed into meaningful information, it may be immensely valuable to share that information with other countries in the region. Dissemination of this type of information may aid in the interpretation of regional events and helps foster better, lasting public health collaborations. SAGES includes tools for two-way communication between public health officials and graphics that are exportable into common image formats. Each SAGES user controls the type and level of detail of all information shared with each recipient (‘role-based access’) and also whether the information sharing is manual or automated. [14] (Figure 5) More importantly, the data are collected and stored only by the user, and remain under the sole control of the user at all times.

#### Modeling/Simulation/Evaluation

JHU/APL has sponsored several electronic disease surveillance exercises to train users and test surveillance system features. [15] We have developed a number of methods for developing simulated outbreaks, both natural and man-made, which can then be ‘injected’ into a simulated database for exercise purposes. JHU/APL has experience with agent-based infectious disease modeling for pandemic influenza as well as other techniques for predictive disease modeling. [16] It is our desire to explore possible collaborations between our disease modelers and users of electronic disease surveillance systems such as SAGES.

The purpose of disease surveillance is to direct the expenditure of limited public health resources in a manner that yields the greatest return on investment. [17] Only those diseases with the highest burdens on the population should be followed and surveillance systems should be periodically evaluated to determine their usefulness. [17], [18], [19] At this time, SAGES does not include tools for the automated evaluation of surveillance systems. Several authors have described the evaluation of electronic disease surveillance systems. [9], [17], [18], [19], [20] We intend to perform such evaluations of SAGES surveillance systems and investigate the feasibility of developing automated evaluation tools in the future.

## Results

### SAGES in the Field

**Figure 6:**
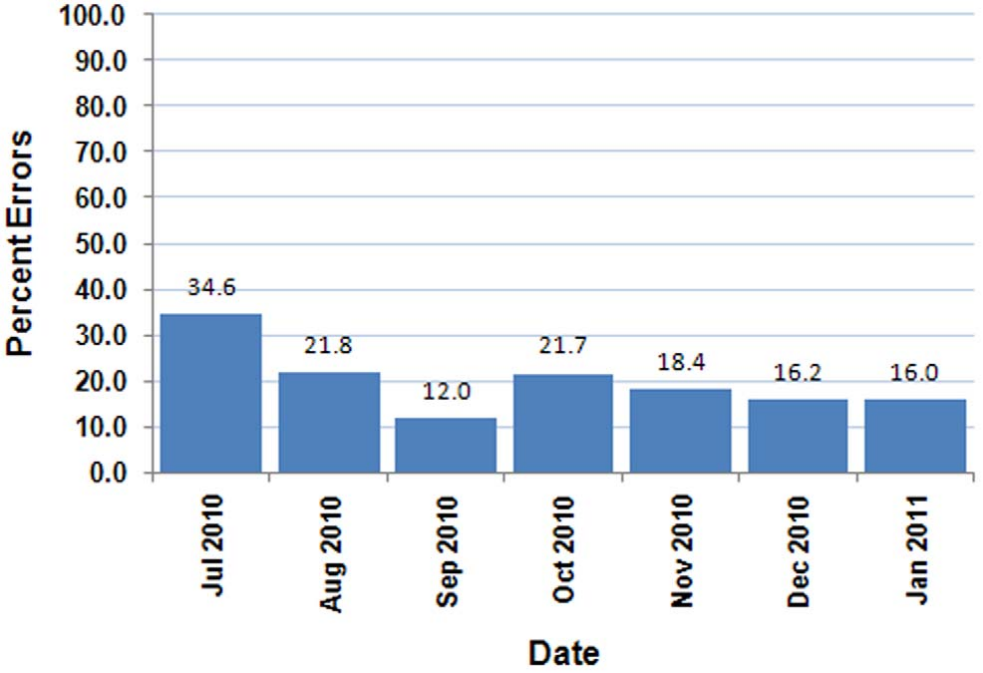
Percent of SMS Protocol Errors by Date. In the Philippines, the percent error of data transmitted via SMS decreased over time.

Pilot activities in Peru and the Philippines have provided valuable insight into how electronic disease surveillance systems can be successful in resource limited environments. The Peruvian system uses IVR technology for data collection. The phone calls needed for IVR are very expensive in the Philippines while SMS text messages cost very little. With that in mind, an existing paper-based fever surveillance system was modified to allow basic information (age, sex, clinic, and symptoms) on patients presenting with fever to be sent to the health department daily via SMS using a standardized texting protocol and abbreviations. The SMS messages are received by a cell phone attached to a computer located at the health department and routed through a simple data cleansing program that sequesters records with incorrect formatting and sends a standard text message to the sender asking them to resend the data. The correctly formatted messages are copied to a database that is then analyzed in EDE. Some clinics quickly adapted to the SMS protocol, while others have found it more difficult to send error-free messages. The percent error decreased over time (Figure 6) suggesting that, with constructive help, most clinics were able to become proficient with the SMS protocol. As of January 2011, approximately 50% of eligible districts are sending daily fever information via text, and the data are being regularly reviewed in EDE. The remaining districts continue to send paper reports or report by phone. The primary reasons given for not using the SMS protocol were lack of personnel or discomfort sending SMS text messages. Focused, repeated training also helped some clinics get started.

## Discussion

### Challenges to Development

The development and implementation of an open-source, electronic disease surveillance system in a resource limited setting faces many challenges. Assuring the smooth interoperability of all software components is sometimes difficult, as is balancing functionality with simplicity for sites with minimal infrastructure. Additionally, finding the appropriate person or group within a country to champion the implementation of a new surveillance system can be fraught with financial, political, territorial challenges. Finally, the open source nature of the software inherently has the consequence of a lack of control of modifications to the software after it is released, potentially leading to incompatible and unverified versions. Conversely, if these changes can be shared in a Wiki-like environment, the entire community using SAGES may benefit from novel improvements.

### Summary

The SAGES project is intended to enhance electronic disease surveillance capacity in resource-limited settings around the world. We have combined electronic disease surveillance tools developed at JHU/APL with other freely-available, interoperable software tools to create SAGES. We believe this suite of tools will facilitate local and regional electronic disease surveillance, regional public health collaborations, and international disease reporting. SAGES tools are currently undergoing pilot testing in locations in Southeast Asia and South America, and will be offered to other interested countries around the world. Opportunities for collaboration are currently being discussed in Central and East African sites as well.
